# Automatic online detection of atrial fibrillation based on symbolic dynamics and Shannon entropy

**DOI:** 10.1186/1475-925X-13-18

**Published:** 2014-02-17

**Authors:** Xiaolin Zhou, Hongxia Ding, Benjamin Ung, Emma Pickwell-MacPherson, Yuanting Zhang

**Affiliations:** 1 Institute of Biomedical and Health Engineering, Shenzhen Institutes of Advanced Technology, Chinese Academy of Sciences, Xili Nanshan, Shenzhen 518055, China; 2 The Key Laboratory for Health Informatics of the Chinese Academy of Sciences at Shenzhen Institutes of Advanced Technology, Shenzhen 518055, China; 3 Department of Electronic Engineering, The Chinese University of Hong Kong, Shatin, N.T., Hong Kong

**Keywords:** ECG, RR interval, Atrial fibrillation, Nonlinear filter, Integer filter, Symbolic dynamics, Shannon entropy

## Abstract

**Background:**

Atrial fibrillation (AF) is the most common and debilitating abnormalities of the arrhythmias worldwide, with a major impact on morbidity and mortality. The detection of AF becomes crucial in preventing both acute and chronic cardiac rhythm disorders.

**Objective:**

Our objective is to devise a method for real-time, automated detection of AF episodes in electrocardiograms (ECGs). This method utilizes RR intervals, and it involves several basic operations of nonlinear/linear integer filters, symbolic dynamics and the calculation of Shannon entropy. Using novel recursive algorithms, online analytical processing of this method can be achieved.

**Results:**

Four publicly-accessible sets of clinical data (Long-Term AF, MIT-BIH AF, MIT-BIH Arrhythmia, and MIT-BIH Normal Sinus Rhythm Databases) were selected for investigation. The first database is used as a training set; in accordance with the receiver operating characteristic (ROC) curve, the best performance using this method was achieved at the discrimination threshold of 0.353: the sensitivity (*Se*), specificity (*Sp*), positive predictive value (*PPV*) and overall accuracy (*ACC*) were 96.72%, 95.07%, 96.61% and 96.05%, respectively. The other three databases are used as testing sets. Using the obtained threshold value (i.e., 0.353), for the second set, the obtained parameters were 96.89%, 98.25%, 97.62% and 97.67%, respectively; for the third database, these parameters were 97.33%, 90.78%, 55.29% and 91.46%, respectively; finally, for the fourth set, the *Sp* was 98.28%. The existing methods were also employed for comparison.

**Conclusions:**

Overall, in contrast to the other available techniques, the test results indicate that the newly developed approach outperforms traditional methods using these databases under assessed various experimental situations, and suggest our technique could be of practical use for clinicians in the future.

## Background

Atrial fibrillation (AF) is recognized as the most common clinically encountered arrhythmia in adults [1], which affects approximately 0.4% of the general population. The prevalence of this tachyarrhythmia increases with age, with less than 1% affected in persons under the age of 60 years and in excess of 6% for those over the age of 80 years [2,3]. Atrial fibrillation is associated with a high risk of stroke, heart disease (e.g., congestive cardiac failure), and cardiovascular mortality [1,4]. There is also a close relationship between AF and obesity [5], obstructive sleep apnea [6], and long-term alcoholism [7], which reciprocally bear cumulative risks for promoting the development of AF [1]. The early identification of AF appears to be crucial for patients with cardiovascular disease, especially for stroke patients to whom the secondary stroke prevention is of primary importance.

Issues relating to clinical significance of rhythm classification and the impetus for improving the accuracy of atrial tachyarrhythmia estimation have motivated the development of innovative computerized AF detectors. Since the early 1980s, a series of sophisticated methods have been investigated to cope with the challenges of AF detection [8-25]. Most of which are based upon two main character traits of this type of arrhythmia shown in a surface electrocardiogram (ECG): (*i*) RR (R-wave peak to R-wave peak) interval irregularity (i.e., chaotic behavior of heart rate variability), and (*ii*) P-wave absence (PWA) or F-wave substitution (i.e., very low amplitude waveforms of odd morphologies) resulting from the abnormal rapid atrial activity (AA). Although P waves or cardiac AA can be an alternative clue in the detection of AF, the absence or presence of P waves are not readily identifiable as various types of high-intensity noise often coexist in ECGs, which may lead to a low degree of predictive accuracy. In addition, the relationship between AA in the surface ECG and the diverse mechanisms of AF has not yet been well delineated [3]. Due to the challenges in detecting AA in ECG measurements, detection techniques based on inferences from RR intervals are preferred to produce relatively robust outcomes [21-23,25].

In this study, a reliable method for the fully automated detection of AF episodes from surface ECGs is proposed. This method comprises of a three-pass procedure. The initial pass, where a RR interval sequence is pre-processed with nonlinear and integer filters, which aims to generate low/high scale reference sequences. The second pass, which aims to obtain a symbolic sequence, where the information of the RR interval sequence is subsequently compressed by the symbolic dynamics with sequences obtained from the initial pass. Finally, Shannon entropy is used in the third pass, to calculate the entropy of the symbolic sequence and thereby discriminate whether or not AF is present in the current cardiac beat. Further methodological insight of present key points on the online analytical processing of measurements through the recursive realization with respect to beat-by-beat classification is discussed in the following sections. Ultimately, we quantitatively investigate the performance of our newly developed technique to that of currently state-of-the-art techniques with four widely used clinical databases under various experimental situations.

## Methodology

### Pre-processing of *RR*_*n*_ series

#### A. Median filter

**Figure 1:**
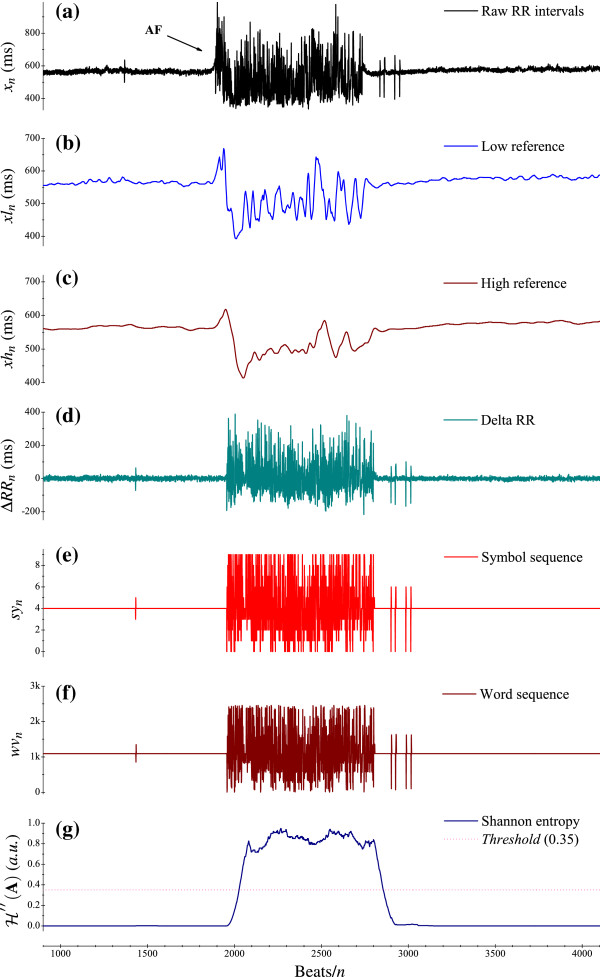
**Example for the application of this method for detecting AF.(a)** Raw RR interval sequence *x*_*n*_; **(b)** Low scale reference *xl*_*n*_; **(c)** High scale reference *xh*_*n*_; **(d)** Difference $\Delta {\text{RR}}_{n}=x_{n}^{\prime }-{\text{xl}}_{n}^{\prime }$; **(e)** The distribution of symbols *sy*_*n*_; **(f)** The relevant word sequence of *sy*_*n*_ in **(e)**, and **(g)** The distribution of SE ${ℋ}^{\prime \prime }(A)$.

A median filter is implemented by windowing the acquired data, ranking the samples in the window, and outputting the median of the sorted samples. Considering a RR interval (*RR*_*n*_) sequence *x*_*n*_, as shown in Figure 1(a), the output *y*_*n*_ of this nonlinear filter is given by,

(1) $$y_{n}=\text{median}\{x_{n-w},\cdots \;,x_{n},\cdots \;,x_{n+w}\}$$

where the window is of a fixed width 2*w*+1. From the perspective of signal processing, the time delay of the median filter is *w*. A window size of 17 is used herein, with a delay of 8 samples. The introduction of a median filter brings about two advantages: (*i*) the suppression of unwanted outliers, which are mostly caused by erroneously detected (or missed) R-wave peaks; (*ii*) to preserve sharp edges (i.e., onsets and terminations of AF episodes) without extensively blurring the context.

#### B. Integer filter for low scale reference

Subsequently, we filter the output *y*_*n*_ of median filter with a low-pass filter of the form

(2) $$H_{l}(z)=\frac{1-z^{-16}}{1-z^{-1}}$$

where, the gain is *Gain*1=16=2^4^, and the intrinsic delay of *H*_*l*_(*z*) is 7.5 samples. This low-pass filter is applied to smooth *y*_*n*_ resulting from the previous median filtering. Another benefit of the low-pass filter is the removal of fluctuations possibly caused by Respiratory Sinus Arrhythmia (RSA) phenomena around the current sample from acquisition. Let *xl*_*n*_ be the output of this filter, as illustrated in Figure 1(b).

#### C. Integer filter for high scale reference

Another low-pass filter *H*_*h*_(*z*) is then applied to the resultant *xl*_*n*_ of the previous low-pass filter *H*_*l*_(*z*),

(3) $$H_{h}(z)=\frac{1-z^{-32}-z^{-64}+z^{-96}}{1-2z^{-1}+z^{-2}}$$

where, the gain is *Gain*2=2048=2^11^, and the relevant delay of *H*_*h*_(*z*) is 47 samples. This low-pass filter is introduced to generate a reference RR sequence of a larger scale, which needs to be exploited in the definition of symbolic series as explained in the following subsection. The resulting output denoted by *xh*_*n*_ is shown in Figure 1(c).

As we have seen, the time delays of *x*_*n*_ and *xl*_*n*_ are −62.5 and −47 samples with respect to *xh*_*n*_, respectively. To ensure synchronization of the filtered data, let $x_{n}^{\prime }$ and ${\text{xl}}_{n}^{\prime }$ denote the corresponding time-delay corrected sequences of *x*_*n*_ and *xl*_*n*_, respectively. Then, $\Delta {\text{RR}}_{n}=x_{n}^{\prime }-{\text{xl}}_{n}^{\prime }$ can be defined as the difference in time delay, seen in Figure 1(d).

### Symbolic dynamics of *ΔRR*_*n*_

The purpose of employing symbolic dynamics is to describe the dynamic behavior of *ΔRR*_*n*_ with respect to *xh*_*n*_. Symbolic dynamics encodes the information as a variation of *RR*_*n*_ to a series with fewer symbols, with each symbol representing an instantaneous state. The implemented thresholds can be defined as: *thre*1=*xh*_*n*_×2^−4^ (with *thre*1=*xh*_*n*_>>4), *thre*2=*xh*_*n*_×2^−3^ (with *thre*2=*xh*_*n*_>>3), *thre*3=*thre*1+*thre*2, *thre*4=*xh*_*n*_×2^−2^ (with *thre*4=*xh*_*n*_>>2) and *thre*5=*thre*4+*thre*1. The mapping function of the symbol transform can therefore be defined as,

(4) $${\text{sy}}_{n}=\left\{\begin{array}{cc} 0 & \;\;\;\text{if}\;\Delta {\text{RR}}_{n}<-\text{thre}4\\ 1 & \;\text{else if}\;\Delta {\text{RR}}_{n}<-\text{thre}3\\ 2 & \;\text{else if}\;\Delta {\text{RR}}_{n}<-\text{thre}2\\ 3 & \;\text{else if}\;\Delta {\text{RR}}_{n}<-\text{thre}1\\ 4 & \;\text{else if}\;\Delta {\text{RR}}_{n}<\text{thre}1\\ 5 & \;\text{else if}\;\Delta {\text{RR}}_{n}<\text{thre}2\\ 6 & \;\text{else if}\;\Delta {\text{RR}}_{n}<\text{thre}3\\ 7 & \;\text{else if}\;\Delta {\text{RR}}_{n}<\text{thre}4\\ 8 & \;\text{else if}\;\Delta {\text{RR}}_{n}<\text{thre}5\\ 9 & \;\;\;\;\text{other cases} \end{array}\right)$$

The raw RR sequence *x*_*n*_ is then quantified into symbol sequence *sy*_*n*_ with specific symbols from the predefined “alphabet” in Eq. (4) (i.e., 0 to 9). Recalling Figure 1(a)-(d) and scanning the distribution of calculated symbols in Figure 1(e), we confirm that most of normal beats are defined as zero symbols, and possible abnormal beats (arrhythmias, e.g., AF) are defined as non-zero symbols by the transform Eq. (4).

**Figure 2:**
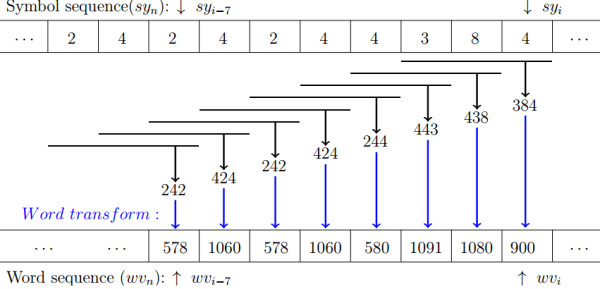
**Schematic illustrating the symbol definition and the word transformation by Eq. (**5**).**

To facilitate the analysis of *sy*_*n*_, the widely used 3-symbol template (i.e., a word consists of 3 successive symbols) is applied to examine entropic properties. The word value can then be calculated by a novel operator as defined below,

(5) $${\text{wv}}_{n}=({\text{sy}}_{n-2}\times 2^{8})+({\text{sy}}_{n-1}\times 2^{4})+{\text{sy}}_{n}$$

where, *sy*_*n*−2_×2^8^ and *sy*_*n*−1_×2^4^ are implemented with *sy*_*n*−2_<<8 and *sy*_*n*−1_<<4, and 0≤*wv*_*n*_≤2457. Figure 2 briefly elucidates the transformation of the symbol sequence with the template and the corresponding word, while Figure 1(f) depicts the word sequence of *sy*_*n*_ shown in Figure 1(e).

### Shannon entropy

Shannon entropy (SE) is a statistical tool that quantifies a time series in terms of the information size. For the sake of completeness, we define the discrete probability space of a dynamic system as **A**=(*A*|*P*). The total number of elements in **A** is *N*. The characteristic elements can then be defined as *A*={*a*_1_,⋯,*a*_*k*_}, as well as the relevant probability set *P*={*p*_1_,⋯,*p*_*k*_}(1≤*k*≤*N*). Each element *a*_*i*_ has probability *p*_*i*_=*N*_*i*_/*N* ($0<p_{i}\leq 1,\overset{k}{\underset{i=1}{\sum }}p_{i}=1$), where *N*_*i*_ is the total number of the element *a*_*i*_ in **A**. Thus, the SE of **A** is defined as [26],

(6) $$ℋ(A)=-\overset{k}{\underset{i=1}{\sum }}p_{i}\underset{2}{\log}p_{i}$$

By Jensen’s inequality, we can prove $ℋ(A)\leq \underset{2}{\log}k\leq \underset{2}{\log}N$ with equality if *p*_*i*_≡1/*Nandk*≡*N* for all *i*. Then, a uniform distribution of ℋ(A) can be expressed as,

(7) $${ℋ}^{\prime }(A)=-\frac{1}{\underset{2}{\log}N}\overset{k}{\underset{i=1}{\sum }}p_{i}\underset{2}{\log}p_{i}$$

where, if *N*≡1, make log2*N*=1. Eq. (7) is also referred to as the normalized entropy, since the entropy is divided by the maximum entropy log2*N*. A coarser version of ${ℋ}^{\prime }(A)$ can be defined as,

(8) $${ℋ}^{\prime \prime }(A)=-\frac{k}{N\underset{2}{\log}N}\overset{k}{\underset{i=1}{\sum }}p_{i}\underset{2}{\log}p_{i}$$

Currently, the dynamic **A** consists of all 127 consecutive word elements from *wv*_*n*−126_ to *wv*_*n*_ (the bin size in this case is *N*=127). By determining the characteristic set *A* and the relevant probability set *P* with these elements, we can thus calculate the SE ${ℋ}^{\prime \prime }(A)$. The presence of AF is then detectable, with the rhythm labeled AF if ${ℋ}^{\prime \prime }(A)$ exceeds a discrimination threshold, and otherwise non-AF, which can be seen in Figure 1(g). We utilize the training database to determine the optimal discrimination threshold by investigating various threshold settings which lie within the range [0.0, 1.0]; the best performing threshold of 0.353 is thus derived and employed for the performance assessment using different testing databases.

### Key issues of online processing

From Eq. (1)–(5) and (8), outwardly, this AF detection technique poses computational challenges. However, these challenges can be overcome by implementing clever recursive algorithms with beat-by-beat, real-time processing.

#### A. Pseudo-recursive median filtering

The median filter in Eq. (1) can be implemented with a so-called pseudo-recursive method: for input *x*_*i*_, we define *S*={*s*_*r*_*↑*:1≤*r*≤2*w*+1} as a sorted array of successive elements from *x*_*i*−2*w*−1_ to *x*_*i*−1_, where the output *y*_*i*_ is obtained by following steps ➊-➎ below, 

➊ A Binary search technique is used to seek out the position *m* of the sample *x*_*i*−2*w*−1_ which will depart from the window (i.e., *s*_*m*_=*x*_*i*−2*w*−1_. Simultaneously, *x*_*i*_ will get into the window);

➋ The Binary search technique is applied again to search for the position *t* at which the input *x*_*i*_ needs to be set (i.e., *s*_*t*_<*x*_*i*_≤*s*_*t*+1_);

➌ From positions *m* to *t*, the current *s*_*r*_ is replaced with the adjacent *s*_*r* ± 1_ (the ’ _±_’ indicates where the element is taken from the right or left, with the ’ _+_’ and ’ _−_’ symbols representing the element to the right and left, respectively);

➍ Replace the element *s*_*t*_ with *x*_*i*_;

➎ Median *s*_*w*+1_ of the updated *S* becomes output *y*_*i*_.

**Figure 3:**
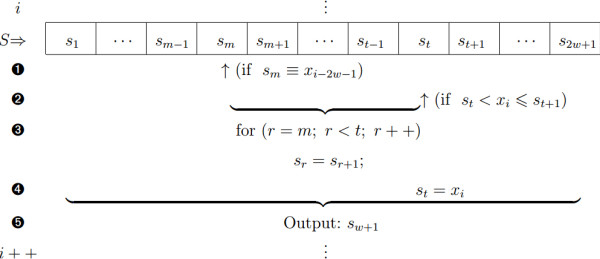
Schema of the pseudo-recursive median filtering (the rightward case).

For the following input *x*_*i*+1_, we repeat steps ➊ to ➎ and obtain the new output *s*_*w*+1_ (i.e., *y*_*i*+1_), as shown in Figure 3, where the sorting utilizes the Binary search technique twice. Comparing our technique with the traditional median filter, the computational complexity can be decreased from approximately *O*(*n*^2^) to *O*(*n*).

#### B. Recursive implementation of integer filters

The recursive implementation (also referred to as the “difference equation”) of the filter *H*_*l*_(*z*) can be expressed as,

(9) $${\text{xl}}_{n}={\text{xl}}_{n-1}+y_{n}-y_{n-16}$$

The above equation, Eq. (9) includes 1 integer addition, 3 integer subtractions as well as 1 integer right-shift operation, when *xl*_*n*_>>4 (as *Gain*1=2^4^) to offset the gain of *H*_*l*_(*z*).

The filter *H*_*h*_(*z*) can then be computed recursively using

(10) $$\begin{array}{ll} {\text{xh}}_{n} & =({\text{xh}}_{n-1}\times 2)-{\text{xh}}_{n-2}\\ & \;+{\text{xl}}_{n}-{\text{xl}}_{n-32}-{\text{xl}}_{n-64}+{\text{xl}}_{n-96} \end{array}$$

where, *xh*_*n*−1_×2 is implemented with *xh*_*n*−1_<<1. The above equation, Eq. (10) consists of 2 integer additions, 8 integer subtractions, 1 integer left-shift operation and 1 integer right-shift operation, when *xh*_*n*_>>11 (as *Gain*2=2^11^) to offset the gain of *H*_*h*_(*z*).

#### C. Mapping the definition of $-\frac{1}{\underset{2}{\log}N}p_{i}\underset{2}{\log}p_{i}$

Investigating the dynamic **A**, we immediately see that each characteristic symbol of each bin *N* may have the probability *p*_*i*_=*i*/*N* (1≤*i*≤*N*, i.e., 1/*N*≤*p*_*i*_≤1). Along these lines, a probability array *PiMap* can be pre-calculated,

(11) $$\begin{array}{ll} \text{PiMap}[127] & =-\frac{\text{Cons}}{\underset{2}{\log}N}\left\{p_{1}\underset{2}{\log}p_{1},\cdots \;,p_{63}\underset{2}{\log}p_{63},\right)\\ & \;\left(p_{64}\underset{2}{\log}p_{64},\cdots \;,p_{127}\underset{2}{\log}p_{127}\right\}\\ & \overset{\left\lfloor \cdot \right\rfloor }{=}\{7874,\cdots \;,71790,71291,\cdots \;,0\} \end{array}$$

where, *Cons*=1000000 is a constant such that decimal floating points can be converted into integers and *N*=127, and $\overset{\left\lfloor \cdot \right\rfloor }{=}$ indicates to take the integer part of each $-\frac{\text{Cons}}{\underset{2}{\log}N}p_{i}\underset{2}{\log}p_{i}$.

Notably, for each cardiac cycle screened, this predefined *PiMap* permits the sole operation by picking the straightforward integer (i.e., *PiMap*[*i*]) from the set *PiMap* in accordance with the index *i* rather than calculating $-\frac{1}{\underset{2}{\log}N}p_{i}\underset{2}{\log}p_{i}$ using arithmetic and logarithmic operations. The use of this predefined calculation significantly decreases calculation times.

#### D. Recursive implementation of ${ℋ}^{\prime \prime }(A)$

We define a buffer array ${\text{nu}}_{wv_{i}}$ (*wv*_*i*_≤2457) to store the number of the *i*th characteristic element *wv*_*i*_ in space **A**. For the input *wv*_*n*_, it will get into **A** (i.e., *wv*_*n*_ will be the rightmost element), and simultaneously the leftmost element *wv*_*n*−127_ will depart from **A**, see Figure 2 for clarity. It is obvious that a variation of SE ${ℋ}^{\prime }(A)$ is purely determined by ${\text{nu}}_{wv_{n}}$ and ${\text{nu}}_{wv_{n-127}}$ in dynamic **A**. Therefore, ${ℋ}^{\prime \prime }(A)$ is calculated recursively by the algorithm below,

where $sh_{n}^{\prime }$ and $sh_{n}^{\prime \prime }$ represent ${ℋ}^{\prime }(A)$ and ${ℋ}^{\prime \prime }(A)$, respectively; ^(∗^ indicates that *PiMap*[*i*]=0 is fixed for the case *i*≡0; and 127000000=*N*∗*Cons*=127×1000000. For the next input *wv*_*n*+1_, steps âž€-âž‚ are again executed to obtain $sh_{n+1}^{\prime \prime }$. From an online processing perspective, the time delays of $sh_{n}^{\prime \prime }$ are 64 and 126.5 samples with respect to *xh*_*n*_ and *x*_*n*_, respectively.

**Figure 4:**
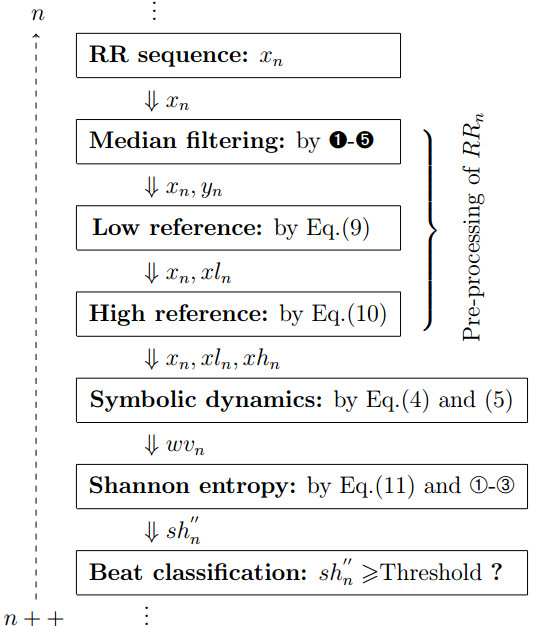
Flowchart of the recursive realization of this detector for beat-by-beat assessing AF.

An architecture of the overall logic of the recursive realization can be seen in Figure 4. By using recursive algorithms, this AF detector consists of several basic operations, such as integer addition/subtraction, integer comparison and integer shifting. In effect, the calculation of $sh_{n}^{\prime \prime }$ and distinguishing the current beat *x*_*n*_, only needs to include 1 multiplication and 1 division lying within $\frac{k}{127000000}\cdot$, together with 1 floating-point comparison between $sh_{n}^{\prime \prime }$ and a threshold. Consequently, a useful computational efficiency can be achieved.

## Materials and evaluation

### Clinical ECG data sets

Performance of this new AF detection method is evaluated using four popular sets of clinical ECGs (the Long-Term AF Database [LTAFDB], the MIT-BIH AF Database [AFDB], the MIT-BIH Arrhythmia Database [MITDB], and the MIT-BIH Normal Sinus Rhythm Database [NSRDB]) [27]. The LTAFDB database is used as the initial training set, while the other three databases are used as the testing sets. The contents of these databases are summarized in Table 1. All reference annotations of the four databases are examined in this study.

**Table 1:** Four publicly-accessible sets of clinical data are selected for evaluation

**Databases**	***f***_***s***_	**Total beats**	**Brief description**
	**(Hz)**	**(AF beats)**	
LTAFDB	128	8996056	It consists of 84 long-term (typically 24 to 25 hours) ECG
		(5326145)	recordings of subjects with paroxysmal or sustained AF.
AFDB	250		It contains 25 long-term (10 hours) ECG recordings of subjects
		1221574	with AF (mostly paroxysmal). Of which raw ECG data of two
		(519687)	records (“00735” and “03665”) are not available, and two
			records (“04936” and “05091”) include many incorrect reference annotations
MITDB	360	109590	It is a collection of 48 half-hour two-lead recordings which were
		(11496)	arrhythmia obtained from 47 subjects and contains affluent
			information, such as AF and AFL
NSRDB	128	1729523	It includes 18 long-term records of subjects. Each recording is
		(0)	about 24 hours in duration. These records had no significant
			arrhythmias detected in this database

### Performance metrics

The performance of our newly developed algorithm and existing methods are investigated in terms of sensitivity (*Se*), specificity (*Sp*), positive predictive value (*PPV*), and overall accuracy (*ACC*),

(12) $$\begin{array}{cc} \text{Se} & =\frac{\text{TP}}{\text{TP}+\text{FN}},\;\text{PPV}=\frac{\text{TP}}{\text{TP}+\text{FP}},\\ \text{Sp} & =\frac{\text{TN}}{\text{TN}+\text{FP}},\;\text{ACC}=\frac{\text{TP}+\text{TN}}{\text{TP}+\text{TN}+\text{FP}+\text{FN}} \end{array}$$

where, for a specific data set, *TP* (true positive) is the number of beats in AF which are correctly detected as AF, *TN* (true negative) is the number of beats in non-AF which are correctly detected as non-AF, *FP* (false positive) is the number of beats in non-AF which are incorrectly detected as AF, and *FN* (false negative) is the number of beats in AF which are incorrectly detected as non-AF. The proportion of beats in true AF which are correctly identified as AF is represented by *Se*, while *Sp* represents the proportion of beats in true non-AF which are correctly identified as non-AF, *PPV* represents the proportion of algorithm results that are true positive, and *ACC* represents the overall accuracy of our method. We consider *Se* and *Sp* as the main metrics, while *PPV* and *ACC* are complementary.

### Results and discussion

**Figure 5:**
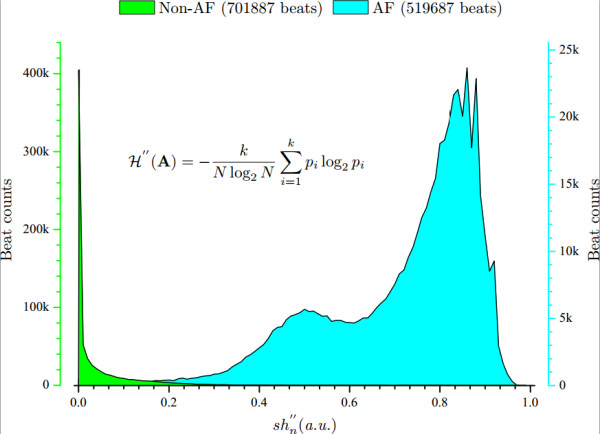
**Histogram distribution of the**${ℋ}^{\prime \prime }(\text{A})$** for annotated AF and non-AF beats of the AFDB database.**

The values of SE ${ℋ}^{\prime \prime }(A)$ for AF (519687 beats) and non-AF (701887 beats) annotations in the AFDB database (a total of 1221574 beats for all of the 25 records) can be seen in Figure 5. It is apparent that ${ℋ}^{\prime \prime }(A)$ discriminates AF well.

**Figure 6:**
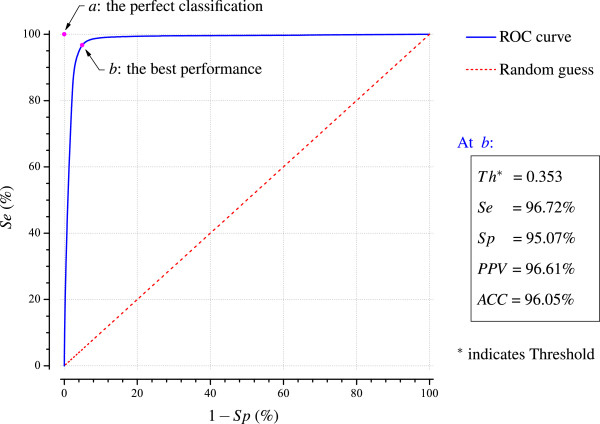
**ROC curve of the training set of LTAFDB database when our method was applied with the various threshold values from 0.0 to 1.0 in increments of 0.001.** Based upon the results portrayed here, the best performing threshold of 0.353 is used for performance assessment.

The receiver operating characteristic (ROC) curves are widely used in the medical field to determine the optimal discrimination threshold for clinical tests. In this work, the LTAFDB database is used as the training set to obtain the optimal threshold for our algorithm. The threshold is tested from 0.0 to 1.0 in increments of 0.001 for the training set, and the values of *Se*, *Sp*, 1−*Sp*, *PPV* and *ACC* are calculated for each threshold setting. Thus we obtain the ROC curve, as shown in Figure 6. In the ROC space of Figure 6, *a* is the point of the perfect classification, at which the *Se* and *Sp* are both equal to 100% and *b* is the point of the best performance of our method on the ROC curve, at which it has the shortest distance to *a*. We can thus determine the parameters at position *b*, where the discrimination threshold is 0.353, and the values for *Se*, *Sp*, *PPV* and *ACC* are 96.72%, 95.07%, 96.61% and 96.05%, respectively. We therefore take the best performing threshold value of 0.353 for quantitative assessment when our method is applied to other three testing databases.

For our newly presented method, the statistical results from the testing sets AFDB, MITDB and NSRDB databases are summarized in Table 2. Specifically, for the AFDB set, the calculated *Se*, *Sp*, *PPV* and *ACC* parameters are 96.89%, 98.25%, 97.62% and 97.67%, respectively. Examining the AFDB^*‡*^ set (^*‡*^ indicates records “00735” and “03665” omitted), the parameters are 96.82%, 98.06%, 97.61% and 97.50%, respectively. For the AFDB^*†*^ set (^*†*^ indicates records “04936” and “05091” omitted), the parameters are 97.83%, 98.19%, 97.56% and 98.04%, respectively. For the MITDB data set, the parameters are 97.33%, 90.78%, 55.29% and 91.46%. It is important to recognize that for MITDB data set, the *PPV* value (55.29%) is low which indicates that many of the positive results are detected as false positives using this testing procedure. Calculating the combined values from these databases, the parameters are 96.89%, 98.27%, 92.30% and 98.03%, respectively for the AFDB+NSRDB set, and 97.53%, 98.26%, 90.09% and 98.16% for the AFDB^*†*^+NSRDB set. For the NSRDB set, the only calculated parameter *Sp* is 98.28%, as there is no manual AF annotation in the NSRDB database.

**Table 2:** Statistical results of this method for three testing databases (at the threshold of 0.353)

**Method**	**Features**	**Year**	**Database**	**Key techniques**	**Results**			
					***Se*** **(%)**	***Sp*** **(%)**	***PPV*** **(%)**	***ACC*** **(%)**
This method	RRI	2013	AFDB		96.89	98.25	97.62	97.67
			AFDB^‡^	Nonlinear	96.82	98.06	97.61	97.50
			AFDB^†^	filter + integer	97.83	98.19	97.56	98.04
			MITDB	filters + symbolic	97.33	90.78	55.29	91.46
			NSRDB	dynamics + SE	NA	98.28	NA	NA
			AFDB+NSRDB		96.89	98.27	92.30	98.03
			AFDB^†^+NSRDB		97.53	98.26	90.09	98.16

^‡^Records “00735” and “03665” omitted.

^†^Records “04936” and “05091” omitted.

‘NA’ indicates not applicable because there is no beat with AF reference annotation in this database.

The existing algorithms for the AF detection are also investigated using the same databases (i.e., the same records and the same reference annotations), and using the same evaluation metrics. Table 3 shows a collection of latest published results from prior literature. The list is not intended to be exhaustive, and more complete investigations are available in [19,28].

**Table 3:** Overview of published results of the existing methods using the same databases

**Method**	**Features**	**Year**	**Database**	**Key techniques**	**Results**			
					***Se*** **(%)**	***Sp*** **(%)**	***PPV*** **(%)**	***ACC*** **(%)**
Lee, *et al*[25]^*^	RRI	2013	AFDB^†^+NSRDB	Sample entropy	97.26	95.91	–	96.14
Huang, *et al*[23]	RRI	2011	AFDB	Histogram+SD analysis+...	96.1	98.1	–	–
			NSRDB		NA	97.9	NA	NA
Lake, *et al*[22]	RRI	2011	AFDB	COSEn	91	94	–	–
Lian, *et al*[21]^*^	RRI	2011	AFDB	Map of RdR	95.8	96.4	–	–
			MITDB		98.9	78.8	–	–
			NSRDB		NA	90.0	NA	NA
Parvaresh, *et al*[20]^*^	AR	2011	AFDB^‡^	LDA classifier	96.14	93.20	90.09	–
Babaeizadeh, *et al*[16]	RRI/AA	2011^⋆^	AFDB^‡^	Markov	87.27^⋆^	95.47^⋆^	92.75^⋆^	–
	(FSA)	2009			92	–	97	–
Couceiro, *et al*[15]	RRI/AA	2011^⋆^	AFDB^‡^	Neural network classifier	96.58^⋆^	82.66^⋆^	78.76^⋆^	–
	(PWA/FSA)	2008			93.8	96.09	–	–
Schmidt,*et al*[14]	RRI/AA	2011^⋆^	AFDB^‡^	Markov+Templete matching+...	89.20^⋆^	94.58^⋆^	91.62^⋆^	–
	(PWA/FSA)	2008						
Tatento, *et al*[13]^*^	RRI	2011^⋆^	AFDB	Kolmogorov-Smirnov test	91.20^⋆^	96.08^⋆^	90.32^⋆^	–
		2001			94.4	97.2	96.0	–
Slocum, *et al*[12]	AA	2011^⋆^	AFDB^‡^	Power percentage	62.80^⋆^	77.46^⋆^	64.90^⋆^	–
	(PWA/FSA)	1992						
Dash, *et al*[11]	RRI	2009	AFDB^†^	RMSSD+TPR+SE	94.4	95.1	–	–
			MITDB		90.2	91.2	–	–
Kikillus, *et al*[10]^*^	RRI	2007	AFDB+NSRDB	Histogram+DIFF.+pNN200	94.1	93.4	–	–

^*^The authors proposed several methods, in which, the method with the best performance is presented here.

^‡^Records “00735” and “03665” omitted.

^†^Records “04936” and “05091” omitted.

^***⋆***^Reinvestigated in [19].

^‘–’^indicates without report. ‘NA’ indicates not applicable because there is no beat with AF reference annotation in this database. See text or relevant literature for abbreviation.

We first introduce the methods based on the variability of RR intervals (RRI) [10,11,13,21-23,25].

Kikillus, *et al*[10] conducted a Markov modeling (MM) technique to identify AF. The calculated test results of *Se* and *Sp* were 94.1% (+2.79%, values in parentheses are the differences between our results and the reported results, hereinafter the same) and 93.4% (+4.87%) for the AFDB+NSRDB database.

The method introduced by Dash, *et al*[11], relies on the combination of the root mean square of successive differences (RMSSD), the turning points ratio (TPR) and SE. The presence of AF using this method was considered if given conditions based on thresholds were satisfied. For the AFDB^*†*^ database, the calculated *Se* and *Sp* values were 94.4% (+3.43%) and 95.1% (+3.09%), respectively; and 90.2% (+7.13%) and 91.2% (-0.42%) for the MITDB set, respectively. When compared to our method with respect to the MITDB set, the *Sp* is slightly better than our method, however, there is an unacceptably lower rate of AF identification *Se*.

Tatento, *et al*[13] presented a novel technique using the Kolmogorov-Smirnov test. By choosing the AFDB data set for evaluation, the calculated *Se*, *Sp* and *PPV* values were 94.4% (+2.49%), 97.2% (+1.05%) and 96.0% (+1.62%), respectively. Other researchers’ re-investigated corresponding values were 91.20% (+5.69%), 96.08% (+2.17%) and 90.32% (+7.30%) [19], respectively.

Lian, *et al*[21] developed an AF detector with its basis centered on the Map of RR intervals versus change of RR intervals (RdR). For the AFDB and MITDB sets, the *Se* and *Sp* values were 95.8% (+1.09%) and 96.4% (+1.85%), 98.9% (-1.57%) and 78.8% (+11.98%), respectively. The calculated *Sp* for the NSRDB database was 90.0% (+8.28%). By comparison, when tested on the MITDB set, the *Se* is slight higher than that of our new method; there is, however, a markedly lower rate of non-AF detection *Sp*.

An attractive approach to AF detection was initiated by Huang, *et al*[23]. It utilized a histogram of *ΔRR*_*n*_ and standard deviation (SD) analysis. The calculated *Se* and *Sp* were 96.1% (+0.79%) and 98.1% (+0.15%), when the AFDB set was assessed. The calculated *Sp* was 97.9% (+0.38%) for the NSRDB database. It provided the closest performance to that of this newly proposed method, as can be seen in Tables 2 and 3.

Lee, *et al*[25] investigated three statistical techniques to determine the presence of AF, and the best performance achieved when Sample entropy was employed. Using the AFDB^*†*^+NSRDB data set, the calculated *Se*, *Sp* and *ACC* were 97.26% (+0.27%), 95.91% (+2.35%) and 96.14% (+2.02%), respectively.

Parvaresh, *et al*[20] evaluated three classifiers for AF screening by using autoregressive modeling (AR). Within this method, AR coefficients of 15-second segments of ECGs were taken as features. When tested with the AFDB^*‡*^ set, the best performance occurred at the so-called LDA classifier: the calculated *Se*, *Sp* and *PPV* were 96.14% (+0.68%), 93.20% (+4.86%) and 90.09% (+7.52%), respectively.

Slocum, *et al*[12] published a method based on the reference of AA. The frequency spectrum analysis (FSA) of the remainder generated by canceling the ventricular activity from the surface ECG was applied for differentiating rhythms. Due to the lack of a constant phase relationship between the atrial and ventricular activities, the performance of this type of technique is not high. The AF detection method based only on AA showed inferior performance as can be clearly seen from the Table 3: evaluated on the AFDB^*‡*^ set, the calculated *Se*, *Sp* and *PPV* values were 62.80% (+34.02%), 77.46% (+20.60%) and 64.90% (+32.71%), respectively [19].

**Figure 7:**
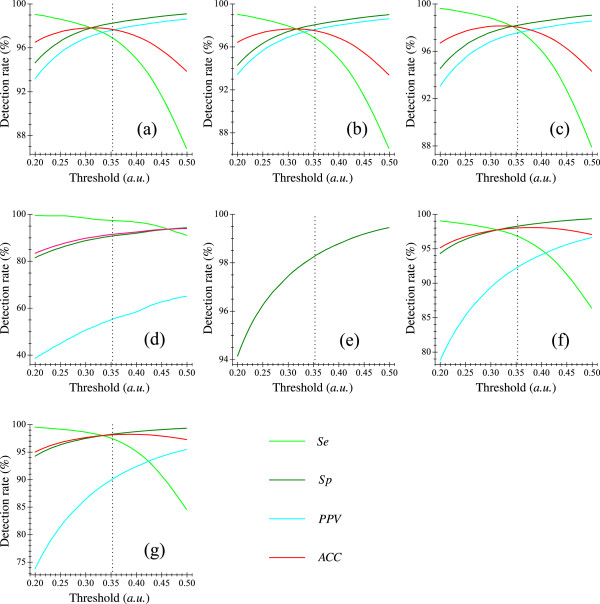
**Distributions of *Se *, *Sp *, *PPV * and *ACC * with respect to various threshold settings when our method was applied to different testing sets.(a)** Results of the AFDB set; **(b)** Results of the AFDB^*‡*^ database (^*‡*^ indicates records “00735” and “03665” omitted); **(c)** Results of the AFDB^*†*^ database (^*†*^ indicates records “04936” and “05091” omitted); **(d)** Results of the MITDB database; **(e)** Results of the NSRDB database; **(f)** Results of the AFDB+NSRDB database and **(g)** Results of the AFDB^*†*^+NSRDB database.

Other methods that take advantage of multiple character traits (i.e., RRI/AA and FSA/PWA) have also been developed [14-16], and were re-investigated in [19]. Recent data consistently indicates these techniques have relatively lower performance, as can be seen in Table 3. The accuracy of multi-feature (or only AA feature) based techniques have been limited by practical challenges encountered in the reliable determination of AA (and/or PWA). Currently, a common rule of thumb is that, as a whole, the sole RRI based techniques are likely to yield better results than those rely on making inferences from multi-feature (or only AA feature) of the surface ECG, since the R-wave peak is the most prominent characteristic trait of an ECG recording and the least susceptible to various kinds of noise.It can be frequently difficult to determine a perfect discrimination threshold for AF episode classification, and it is therefore worth performing further analysis to determine whether the varying discrimination threshold settings significantly influence the performance of our method. For the testing databases, the performance of our method is investigated at various threshold settings. Discrimination threshold values from 0.20 to 0.50 in increments of 0.001 are tested for each of the data sets. Plots of the corresponding results can be seen in Figure 7, where we clearly see that our new method is, preferably performed with the threshold ranging from 0.30 to 0.36. It is sufficient to select a random threshold in this range to investigate the performance of this method. Therefore, the calculated best performing threshold value (i.e., 0.353), derived from the ROC curve of the training set (i.e., LTAFDB database), is appropriate for performance evaluation.

In summary, the results of this study demonstrate that the combination of nonlinear/linear integer filters, symbolic dynamics and SE yields a robust detector. This new detector exhibited a higher detection rate than previous methods. This could possibly lead to incorporation into computerized ECG interpretation systems to improve the reliability of arrhythmia classification.

### A special issue on computational complexity

The computational time of our method is also investigated. Our technique is implemented using the C++ programming language. Table 4 displays the computation time taken by our method while testing with the available databases. Detailed information of the Desktop test environment can be seen in the footnote of Table 4. The computation time of our method is significantly less than the total duration of all records in each database, which indicates that the time consumption is negligible: typically about 0.116 seconds per 24 hours of data processed. Larburu, *et al*[19,28] investigated a variety of existing methods processed on a computer server and they concluded that the method proposed by Cerutti, *et al*[29] had the lowest computation time of approximately 0.36 seconds per 1 hour of data processed (using the AFDB^*‡*^ set, the relevant *Se*, *Sp* and *PPV* were 96.10% (+0.72%), 81.55% (+16.51%) and 75.76% (+21.85%) [19], see [19,28,29] for details). This implies that our method is especially suitable in real-time, long-term ECG monitoring. In addition, Big data is coming of age; our newly developed method shows promise to be of practical use.

**Table 4:** The computation time of the processing of this method

**Databases**	**Signal duration (sec)**	**Computation time (sec)**^§^
LTAFDB	6970560 (1936.27 hours)	11.09
AFDB	917052.96 (254.74 hours)	1.445
AFDB^‡^	843688.72 (234.36 hours)	1.353
AFDB^†^	843688.72 (234.36 hours)	1.406
MITDB	86666.67 (24.07 hours)	0.116
NSRDB	1574976 (437.49 hours)	1.825
AFDB+NSRDB	2492028.96 (692.23 hours)	3.258

^‡^Records “00735” and “03665” omitted.

^†^Records “04936” and “05091” omitted.

^§^Desktop test environment: (a) hardware: Intel Pentium(R) Dual-Core E5800(3.20GHz)/DDR3 RAM (2GBytes,800MHz)/ HDD(7200rpm); (b) software: WINDOWS XP Professional/mingw32-g++/C++. The computation times are the average values of 100 trials, and they include the time consumption for importing raw data from the HDD into the RAM.

## Benefits and limitations

In this study, we use a discrimination threshold of 0.353 for AF classification. Of note, from Figure 7, increasing in threshold value improves *Sp* but decreases *Se*. By contrast, the decreasing in threshold values improves *Se* but decreases *Sp*. A compromising solution is thus necessary, and this makes it easy for one to apply specific threshold settings to the concrete application. In spite of this, comparing the latest detection methods when testing with each database, we confirm that a discrimination threshold of 0.353 is adequate to permit better performance of this new method under various situations.

It is commonly asserted and accepted that there is a great deal of time-consuming routines involved in the assessment of AF due to the statistical analysis of irregular/chaotic arrhythmia characteristics. Dramatic benefits can be achieved with the implementation of this AF detector through properly designed recursive algorithms as well as a novel predefined set $-\frac{1}{\underset{2}{\log}N}p_{i}\underset{2}{\log}p_{i}$ for the calculation of ${ℋ}^{\prime \prime }(A)$, which may markedly reduce computational complexity.

The bin size *N* was set to 127 in this study because a small quantity of words inside a small bin (≪*N*), in general, might indeed reduce the accuracy of estimating the word *wv*_*n*_ probability distribution [30]. However, for sporadic AF episodes of relatively short duration (e.g., ten seconds), it might incur false negative detection, and this may be a potential limitation. In this regard, it is an inherent technical difficulty that needs to be overcome in the future, though AF episodes of very short duration are rare in practice. Nevertheless, it is essential to remember this limitation.

Once again, as stated in the previous section, a small *PPV* calculated from the MITDB database implies that this newly proposed approach needs to be further refined towards a universally applicable method.

## Conclusions

As currently available techiniques are only modestly effective in AF episode screening, we developed a fully automated detection method which aims to fulfill two essential needs: (*i*) earlier real-time identification of AF, and (*ii*) higher reliability of detection. Therefore, with a method available elsewhere for real-time R-wave detection [31], this newly proposed method could be used in intensive care units. The online realization is easy to implement and is computationally attractive as it consists of only several basic operations such as integer addition/subtraction, integer left/right -shifting, integer comparison, and multiplication and division lying only within $\frac{k}{127000000}\cdot$, as well as 1 floating-point comparison between ${ℋ}^{\prime \prime }(A)$ and the threshold for the rhythm classification. Several state-of-the-art methods have been briefly reviewed, along with their methodologies and detection accuracy. Our new method is evaluated and compared with these existing methods using the LTAFDB, AFDB, NSRDB, and MITDB databases under various situations. We have also presented explicit tables for quantitative assessment of the performance and computation times. Collectively, our results suggest that this AF detector outperforms the existing methods with respect to the performance metrics *Se*, *Sp*, *PPV* and *ACC*. It is also worth emphasizing that a few reference annotations of these data sets are themselves imprecise, just as in the AFDB set. Therefore, extensive sets of exact reference annotations are still needed for investigation.

## Appendix

Please visit the “https://onedrive.live.com/?gologin=1&amp;mkt=zh-CN#cid=498A9A3CCEE3B366&amp;id=498A9A3CCEE3B366%21132” for the compiled C++ dynamic link library files or contact the author for them.

## Competing interests

The authors declare that they have no competing interests.

## Authors’ contributions

XZ developed the algorithm and drafted the manuscript. HD collected the clinical ECG data and revised the manuscript. BU, EP and YZ provided suggestions and comments as well as much help in revising the manuscript. All authors read and approved the final manuscript.
